# Atypical presentation of opioid withdrawal, an effect of adulteration

**DOI:** 10.1186/1471-2334-12-S1-P76

**Published:** 2012-05-04

**Authors:** Supriya Kumar Mondal, Biswadeep Borthakur, Kamala Deka, Sabita Dihingia, Dhruba Jyoti Bhuyan

**Affiliations:** 1 Central Institute of Psychiatry, Ranchi, Jharkhand, India; 2 A.R.T Centre, Assam Medical College, Dibrugarh, Assam, India; 3 Jorhat Medical College, Jorhat, Assam, India; 4 AMCH, Dibrugarh, Assam, India

## Background

Brown sugar is the impure form of di-Acetyle Morphine with comparable pharmacological effect and withdrawal symptoms. Recent observation regarding the atypicality withdrawal symptomatology in opioid dependants stimulated the present study.

## Purpose

To study the abuse pattern and symptom profile in withdrawal state of brown sugar abusers.

## Methodology

Consecutive sampling method was used to collect patients with opioid dependence according to DSM IV TR. Abuse pattern was assessed through semi-structure proforma, withdrawal symptoms through clinical opioid withdrawal scale and also chemical analysis of the drug.

## Results

Among patients 43.396% had seizure, 26.086% developed confusion after seizure and 17.391% experienced psychotic symptoms. Longer duration and larger quantity of substance abuse leads to higher complications. Seizure episodes occurred between 11 to 92 hrs of last intake with a median of 30 hrs. The seizure frequency had strong correlation with daily doses (β-0.697) and frequency (r-0.527) but is weakly correlated with withdrawal severity (r-0.425). Chemical analysis of illicit drug revealed that caffeine constitutes greater proportion and opioid like substance a minor quantity.

## Conclusion

Complications like seizure, delirium and psychosis are common in withdrawal. Complication is higher among high quantity and high frequency users. Delirium and psychosis might be a complication of seizure. Adulteration with toxic substance might be a cause for atypical symptoms which leads to a life threatening condition and warrants preventive cure from such illicit drug as opium substitution therapy.

